# Distinguishing between Exploratory and Confirmatory Preclinical Research Will Improve Translation

**DOI:** 10.1371/journal.pbio.1001863

**Published:** 2014-05-20

**Authors:** Jonathan Kimmelman, Jeffrey S. Mogil, Ulrich Dirnagl

**Affiliations:** 1 Studies of Translation, Ethics, and Medicine (STREAM), Biomedical Ethics Unit, Experimental Medicine, McGill University, Montreal, Quebec, Canada; 2 Department of Psychology and Alan Edwards Centre for Research on Pain, McGill University, Montreal, Quebec, Canada; 3 Departments of Neurology and Experimental Neurology, Center for Stroke Research Berlin, and Excellence Cluster NeuroCure Charité – Universitätsmedizin Berlin, Berlin, Germany; 4 German Center for Neurodegeneration Research (DZNE), partner site Berlin, Germany; 5 Center for Cardiovascular Diseases (DZHK), partner site Berlin, Germany; University of Leicester, United Kingdom

## Abstract

Preclinical researchers confront two overarching agendas related to drug development: selecting interventions amid a vast field of candidates, and producing rigorous evidence of clinical promise for a small number of interventions. We suggest that each challenge is best met by two different, complementary modes of investigation. In the first (exploratory investigation), researchers should aim at generating robust pathophysiological theories of disease. In the second (confirmatory investigation), researchers should aim at demonstrating strong and reproducible treatment effects in relevant animal models. Each mode entails different study designs, confronts different validity threats, and supports different kinds of inferences. Research policies should seek to disentangle the two modes and leverage their complementarity. In particular, policies should discourage the common use of exploratory studies to support confirmatory inferences, promote a greater volume of confirmatory investigation, and customize design and reporting guidelines for each mode.

## Abstract 2

Kimmelman and colleagues argue that the key to improving preclinical research lies in distinguishing between two different modes of research: exploratory vs. confirmatory.

## Introduction

The past few years have witnessed growing consternation over the way researchers perform and report preclinical investigations of new drugs. The vast majority of drugs advanced into trials never recapitulate safety and efficacy observed in animal models, and these failures exact a heavy toll on trial volunteers, the research enterprise, and health care systems via higher drug prices. Because many preclinical studies poorly address internal validity threats [1], fail attempts at replication [2], are not published [3], or provide exaggerated estimates of clinical utility, numerous stakeholders are urging reforms in the way preclinical research is performed [4].

We would like to offer a cautionary perspective on these initiatives. We suggest that the ostensibly poor performance of many preclinical studies may in fact reflect strengths and intrinsic properties of what we call “exploratory investigation”—roughly, studies aimed at generating robust pathophysiological theories of disease. Policies aimed at improving translation should strive to preserve the extraordinary power of exploratory studies, which represent the majority of preclinical studies [5], while promoting a separate mode of clinical trial-like preclinical research, which we call “confirmatory” studies—that is, studies aimed at demonstrating strong and reproducible treatment effects in relevant animal models. We close by describing some ways of capitalizing on the complementarity of the two modes.

## Exploratory Versus Confirmatory Research

Clinical translation of novel interventional strategies confronts two overarching challenges. First, researchers must negotiate a virtually unbounded landscape of potential targets, drugs, doses, and treatment regimens. A key task is to develop the theories, measurement techniques, and evidence for selecting a manageable number of interventions to carry forward. Second, clinical development is enormously expensive and exposes patients to unproven and possibly toxic interventions. Another key task of preclinical research is thus to produce evidence that is sufficiently compelling to warrant the economic and moral costs of clinical development.

Overcoming these two challenges necessitates different modes of investigation. The first set of challenges is best met by studies that operate in the *exploratory* mode. We use “exploratory” to capture something broader than what is generally meant in statistics. In our conception, exploratory studies will aim primarily at developing pathophysiological theories that enable pursuit of different approaches. Exploratory studies tend to consist of a package of small and flexible experiments using different methodologies, including molecular and cellular analyses. These individual experiments may or may not employ inferential statistics. Exploratory studies are often driven by a series of hypotheses that are either loosely articulated or that evolve over the course of sequential experiments. Often, exploratory studies include tests of an intervention's efficacy against disease in live animals as a way of validating the pathophysiological theories (“efficacy studies”). Neither the sequence of individual experiments in exploratory studies, nor details of their design (including sample size, since effect sizes may be unknown), is necessarily established at the outset of investigation.

The second set of challenges is best overcome by studies that operate in a *confirmatory* mode. Such studies will resemble adequately powered clinical trials, and consist mainly of “efficacy studies” that use rigid and pre-specified designs, *a priori* stated hypotheses, prolonged durations, and the most clinically relevant assays and endpoints available. These studies aim less at elaborating theories or mechanisms of a drug's action than rigorously testing a drug's clinical potential and restricting the advance of ineffective interventions advanced into clinical testing. Exploratory studies are a complement to confirmatory studies in that the former generates precisely articulated hypotheses about drug effects that can be put to “crucial testing” in the latter before clinical development.

Currently, the vast majority of preclinical studies more closely resemble exploratory studies, although a small but growing number of studies operate in a confirmatory mode. These different orientations carry important imperatives for the design, reporting, error tendencies, and application of preclinical studies. What may be an inferential strength for exploratory study can be a hindrance or even a fatal flaw for confirmatory studies and vice versa. Policies and practices aimed at improving clinical translation should recognize at least four major contrasts between the two modes of investigation.

## Implications for Design and Valid Interpretation

The first difference has already been noted: whereas exploratory studies should mainly aim at deriving or testing theoretical claims, confirmatory studies should test clinical utility of new interventions. Since theories are not directly observable, they are tested by assembling corroboratory evidence across different lines of experimentation. This theoretical orientation in preclinical research is reflected in the fact that a good part of the acreage in publications is devoted to molecular or cellular analyses (e.g., gene expression, immunohistochemistry, electrophysiology), not efficacy studies. Spreading proof across different lines of experiment—a process called “conceptual replication” [6]—has several consequences for predictive value. On the one hand, threats to the validity of theoretical claims driving a preclinical study are mitigated—though not eliminated—by conceptual replications. On the other hand, therapeutic claims arising from efficacy studies contained in the exploratory package will be prone to larger random and systematic variation: such studies invest less in any single experiment, and therefore employ smaller sample sizes and less fastidious designs. In contrast, because confirmatory studies “bet the house” on a single, pivotal efficacy study and measurement technique, there is more at stake scientifically in minimizing random and systematic error.

Second, whereas exploratory studies should place a premium on sensitivity (i.e., detecting all strategies that might be useful), confirmatory studies should be more concerned with specificity (i.e., excluding all strategies that will prove useless in clinical trials). This is because the task of exploration is to catch a small number of promising theories, targets, compounds, doses, or variants of a target indication against a large field. However, in many areas of drug development, the prior probability of discovering useful strategies is extraordinarily low. This means that even in the ideal, where exploratory studies have very high sensitivity *and* specificity, most candidates that are declared promising will represent false positives. Since there are large financial and human costs for advancing these false positives into trials, the task of confirmation is to eliminate “false positives” that are captured in exploration. Further, the agonizingly low positive predictive value of exploratory studies may have as much to do with base rates as it does with bias.

Third, use of small sample sizes for efficacy experiments contained in exploratory studies may lead to large random variation that produces the appearance of bias even in its absence. This dynamic, known as the “winner's curse” [7], reflects the fact that research in the exploratory mode will often test many different strategies in parallel, and this is only feasible if small sample sizes are used. As a consequence of random variation alone, some experiments will produce larger effects that regress to the mean if replicated. In contrast, confirmatory studies should employ sufficiently large sample sizes as to minimize the effect of random variation, such that dwindling effect sizes on replication may be symptomatic of publication bias rather than natural regression.

Last, exploratory studies often involve testing interventions alongside techniques used to measure their effects. In contrast, methods should be well established when an intervention is tested in confirmatory studies. Assays for testing pathophysiological responses, or the probative value of biomarkers, or skills for performing a behavioral test may be still in development at the point of exploratory investigation. One example of this is uncertainty surrounding techniques for testing drugs that target cancer stem cells. Here, standard assays for testing the clinical promise of cancer drugs are almost useless, yet there is little consensus about which assays to use instead [8]. Another example might be where a graduate student conducts experiments before having mastered the requisite manual skills. As a consequence of uncertainty surrounding measurement, exploratory researchers encounter difficulty discriminating informative and uninformative findings: “positive” findings may be attributable to assay artefacts; “negative” findings may reflect defects in the measurement tools, choice of the wrong treatment regimen, or suboptimal experimenter skill. Since the value of uninformative findings for the broader research community is limited, the absence of firm rules for discrimination legitimately confounds decisions about what findings to publish and how to interpret them. Any blanket proscription against “hiding” data risks obscuring truly interesting findings amid a large volume of studies that the experimenter knows to be uninformative to the broader research community: “practice runs,” experiments on miscalibrated instruments, or findings using methods that are later discovered to be error prone. On the other hand, where researchers have grounds for confidence in the regimens for testing, nonpublication of negative findings represents a demonstrable breach of scientific integrity. This will tend to be a much greater concern in confirmatory testing, since measurement techniques tend to be more established in that setting.

In sum, there are many factors that explain why preclinical studies are prone to producing “false positives” or outcome patterns that give the appearance of bias. Yet to some degree, these reflect strengths of exploratory research, such as its ability to narrow the field of intervention candidates using an economy of resources, to select among myriad pathophysiological theories, and to hone techniques of measuring clinical promise. These are necessary precursors to the sorts of rigorous confirmatory experiments that should be used to justify clinical development.

## Improving Design and Interpretation of Preclinical Research

Though some of the above contrasts may appear obvious to anyone with a basic understanding of statistics and experimental design, they are not adequately reflected in many reforms urged by critics of preclinical research—e.g., calls for using larger sample sizes, “gold standard” animal models, or independent replication [9],[10]. Some proposals entail non-trivial burdens such as restructuring laboratory practices, writing up and/or depositing inconclusive findings, or using larger sample sizes, and hence undermine the economy of exploratory activities. Reforms are more likely to have a transformative impact on drug development if researchers can capitalize on the complementary properties of both exploratory *and* translational studies, and tailor study design, reporting, and application of findings accordingly. To that end, we offer three sets of recommendations.

First, all protocols and publications should pre-specify whether they are “exploratory” or “confirmatory” studies, with the latter category reserved for studies that aim at demonstrating promise of clinical utility for an intervention. We note that other commentators have made similar calls [11],[12]. Journal editors and funding agencies should promote this demarcation by requiring it for submitted manuscripts and grants. Standards for review should then hinge on the way investigators classify studies. For instance, confirmatory studies should be held to internal and construct validity standards similar to those used in clinical trials: studies should address confounders like sample or observation bias, use pre-specified statistical analyses, match the experimental design to the conditions where findings are expected to be applied, and report findings in ways that enable meaningful interpretation by non-experts. Large sample sizes, fastidious experimental conditions, and conservative statistical analyses may be counterproductive for exploration. Instead, exploratory studies should be evaluated on the basis of whether findings using disparate and methodologically sound lines of investigation are coherent and fecund.

Second, the research community should devise mechanisms for coupling confirmatory studies to exploratory ones. As noted above, only a small minority of preclinical studies are put to confirmatory testing. Once intervention strategies are discovered in exploration, those wishing to launch clinical development should be expected to run, or at least reference, stand-alone confirmatory studies before launching trials [10],[13]. One way of promoting this would be for oversight bodies—Research Ethics Boards, public funding agencies, and regulators—to condition approval of any trial delivering putatively active drug doses on positive preclinical confirmatory studies. Like clinical trials, such studies should prospectively register, adhere to (and preferably publish) protocols, and report findings according to standards and regardless of effect sizes. As human trial findings are much more informative when they are embedded within a web of related findings [14], medical journals should require that investigators deposit confirmatory preclinical findings when they accept for publication trials involving efficacy primary endpoints.

Third, many recommendations and mechanisms for improving preclinical study design are mainly suited for confirmatory studies. Some recommendations—like calls for more regular replication or simple measures to reduce factors like observer bias (e.g., randomization and assessor blinding) —are sensible across both modes of investigation. Others seem more suited for confirmatory studies and may be counterproductive for exploratory studies. Use of larger sample sizes and prospective registration, for example, involve additional investments, infrastructure, and compliance burdens. The former means sacrifice of more animals than necessary to identify promising strategies. The latter would be very taxing for researchers, since public disclosures early on in a research program would invite free-riding; moreover, registration of exploratory studies offers little to a research community if researchers themselves have significant doubts about measurement techniques. Perhaps the largest validity threats in exploratory research reside not in efficacy studies, but in the withholding of findings that disrupt the coherence of theoretical claims, in the assembly of theories that build on a series of falsely positive experimental results, or in the nonperformance of replication experiments because of insufficient incentive. The research community has much to gain from guidelines and mechanisms that specifically address such tendencies in exploration. One place to start would be to establish data ontologies and databases for deposition of exploratory findings so that discordant findings can be accessed. Another would be the creation of mechanisms that encourage confirmatory studies. For example, several journals now solicit bids for replication studies from the research community, guaranteeing the winning bidder publication on successful completion of the study. Others maintain “results blind” publication categories, where reviews are based on a submitted protocol rather than effect sizes [15],[16]. Journals might also encourage researchers to deposit replications or experiments that are discordant with published exploratory studies, but that are insufficient to constitute a new paper, by creating a section for very short research reports that consist of a single experiment or an attempted replication.

According to influential accounts of the research process, science flourishes best when researchers pursue different agendas, harboring different biases [17]. Preclinical research, in particular, entails two complimentary agendas: one is to narrow a large field of potential therapies by refining pathophysiological theories of disease, and the other is to generate reliable evidence of a therapy's clinical utility in a proxy species. Each encounters different constraints and validity threats. The key to improving preclinical research is devising practices that leverage one to the advantage of the other.
